# Local hyperthermia therapy: a novel strategy turning white fat brown

**DOI:** 10.1093/lifemedi/lnac005

**Published:** 2022-06-24

**Authors:** Lingyan Xu, Yu Li, Toren Finkel, Xinran Ma

**Affiliations:** Shanghai Key Laboratory of Regulatory Biology, Institute of Biomedical Sciences, School of Life Sciences, East China Normal University, Shanghai 200241, China; Shanghai Key Laboratory of Regulatory Biology, Institute of Biomedical Sciences, School of Life Sciences, East China Normal University, Shanghai 200241, China; Aging Institute, University of Pittsburgh School of Medicine/University of Pittsburgh Medical Center, Pittsburgh, PA 15219, United States; Shanghai Key Laboratory of Regulatory Biology, Institute of Biomedical Sciences, School of Life Sciences, East China Normal University, Shanghai 200241, China

Obesity, manifested as the excess accumulation of fat in adipose tissues and other metabolic organs, is a major risk factor for metabolic diseases. As a newly discovered adipose tissue, beige fat is well known for its high functional flexibility of behaving either similar to white fat or to brown fat, depending on systemic energy demands. Upon cold stimuli or β-adrenergic signaling activation, beige fat undergoes a process of “white fat browning” characterized by reprogramming of a set of metabolic genes for thermogenesis and energy expenditure. Although controversy remains, it is recognized that beige fat exists in human adults supraclavicular and paraspinal regions etc., depending on tissue depth. Beige fat can burn considerable amount of glucose and lipid and dissipate these fuels as heat, which represents an attractive strategy for combating obesity and metabolic diseases. Thus, scientists are striving to pursue novel and safe targets and strategies for beige fat activation.

Previous attempts using β3-adrenergic receptor agonists to mimic cold stimulation for beige fat activation encountered various problems, including losing long-term efficacy or causing adverse effects [1] . Intriguingly, not unlike cold treatment, whole-body hyperthermia therapy (HT), i.e. hot tub bathing, sauna and heat blanket wrapping, has been shown to also render metabolic benefits in human and in rodents [2, 3], but the responsive tissues and mechanism underneath are largely unknown. Importantly, HT process involves sustained whole body exposure in a heated environment, which increases core temperature as well as sympathetic tone, thus may lead to heightened risks of cardiovascular diseases [4].

Adipocytes can sense cold temperature in a cell-autonomous way [5]. Using ERthermAC, a newly developed thermosensitive fluorescent dye measuring cellular heat production, we revealed the interesting phenomenon that beige adipocytes also respond to hyperthermia and enhance their thermogenesis in a cell autonomous way. This inspired us to further explore whether local hyperthermia specifically targeting beige fat (a method we termed local hyperthermia therapy, LHT) could induce thermogenesis *in vivo*. If feasible, LHT in beige fat could circumvent side effects of whole-body HT and lead to increased mechanistic understanding. It is fortunate that our lab is adjacent to a nanomaterial laboratory. During a casual chat with our neighbor, we learned that a kind of nanoparticle generated with polyethylene glycol (PEG)-crosslinked polydopamine (PDA) is capable of producing heat at a precise temperature upon adjustable near-infrared (NIR) light induction. It is even better that this material has high bio-compatibility, as well as long-time and wide application for cancer treatment in the nanomaterial field.

Having this suitable avenue for LHT, we sought to test the potential metabolic effects of LTH in rodents. Of note, LHT (injecting PEG-PDA nanomaterial into mice iWAT and making it emit heat of 41°C ± 0.5°C by NIR illumination) induced strong heat induction and increased thermogenic gene programs in beige fat both acutely and chronically in mice. Interestingly, we demonstrated that LHT three times a week for 10 weeks effectively reduced body weights and fat mass, increased cold tolerance and energy expenditure, improved insulin sensitivity and reduced hepatic steatosis in mice fed a high-fat diet. In addition to its protective role in the process of obesity, LHT also treated obesity and metabolic dysfunctions effectively in obese mice. Critically, as a proof of principle, we tested LHT in human subjects by applying a heat source of controlled temperature to the supraclavicular area of human volunteers, an area reported to be enriched for beige fat. After the completion of LHT, we found sustained and enhanced heat production in this region, suggesting that LHT could also stimulate beige fat in humans.

With the discovery of phenotype comes the deciphering of mechanisms. Naturally, the first thought came to mind is to screen classic factors responding to heat, like heat shock factor 1 (HSF1) and Transient receptor potential vanilloid 1 (TRPV1). Using siRNAs, we found that LHT on beige adipocytes at 41°C ± 0.5°C requires HSF1, but not TRPV1, which is consistent with a threshold of 43°C for TPRV1 activation. HSF1 is a master regulator of the heat-shock response, a powerful adaptive mechanism that enables organisms to cope with a wide variety of environmental stresses, including heat, via heat shock proteins (HSPs) dependent and independent manners. We thus generated a HSF1 fat-conditional knockout (HSF1-FKO) mouse model by crossing Adiponectin-Cre mice with HSF1-Loxp mice to examine whether LHT’s effects were mediated by HSF1 in adipose tissues. We found that HSF1-FKO mice were prone to diet-induced obesity and metabolic dysfunctions. They also featured reduced thermogenic capacity and energy metabolism. More importantly, HSF1-FKO mice were resistant to LHT-induced metabolic benefits, while delivering an AAV-mediated active form of HSF1 in iWAT mimicked the metabolic benefits of LHT by promoting white fat browning. Together, these genetic experiments suggest that HSF1 is indispensable for the metabolic effects of LHT.

Next, for a more detailed molecular mechanism, we performed ChIP-seq analysis to catalogue the direct target genes of HSF1 in beige adipocytes on a whole genome scale. We found that in addition to classic HSP genes, HSF1 is highly enriched on the heat shock response element of the promoter of a RNA-binding protein Hnrnpa2b1 (A2b1). This regulation was further confirmed by ChIP analysis and luciferase assays. We and others have previously shown that RNA-binding proteins exert critical metabolic functions in various metabolic diseases. Indeed, we found that A2b1 levels were closely correlated with beige fat activities, suggesting the involvement of A2b1 in energy homeostasis. A2B1 overexpression improved metabolic performances with enhanced browning of white fat in mice, while conversely, A2B1 knockdown in beige fat or A2B1 haplo-insufficient in mice led to adverse metabolic phenotypes.

A2B1 regulates RNA levels mainly by three means, RNA splicing, miRNA maturation and mRNA stability. We thus studied how A2B1 impacted energy metabolism. Via RNA-seq analysis and miRNA examination, we were able to exclude the first two possibilities. Subsequently, RNA immunoprecipitation analysis and luciferease assay revealed that A2B1 binds to the 3ʹUTR of key metabolic genes, including Pgc1α and Ucp1, and enhances their mRNA stability. Consistent with these observations, A2B1 overexpression/knockdown increased/decreased mRNA stability of these thermogenic genes, respectively. In addition, A2B1 overexpression rescued HSF1 deficiency-induced mRNA instability, suggesting that A2B1 is a critical downstream effector of HSF1 in beige fat.

Finally, we explored the clinical relevance of HSF1 genetic variants on metabolic traits in over 10,000 participants from the Shanghai Nicheng Cohort and Shanghai Fat Distribution and Disease (FADE) using genome-wide association study and exome-wide association study. We discovered a missense variant p.Pro365Thr (rs78202224) within the HSF1 gene and it is associated with mild but significant decreases in body mass index and serum triglyceride levels, as well as a strong improvements in glucose metabolism. We then performed *in vitro* and *in vivo* studies and showed that this HSF1 variant increases A2b1 transcripts in beige adipocytes and beige fat.

**Figure 1.**
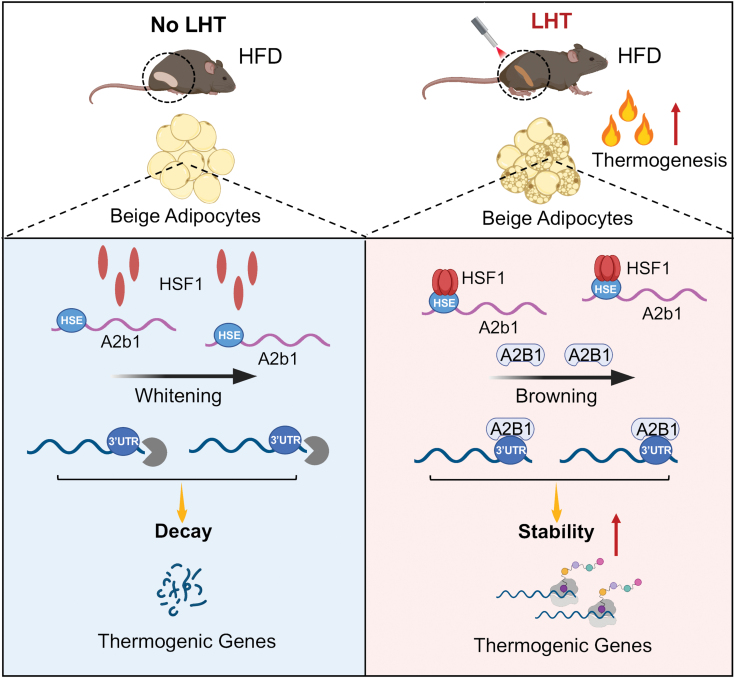
Illustration figure showing that local hyperthermia therapy (LHT) on beige fat leads to activation of HSF1-A2b1 transcriptional axis for enhanced mRNA stability of thermogenic genes.

Overall, the present study revealed LHT to be a promising strategy for beige fat activation and metabolic improvements, meanwhile delineated a new HSF1-A2B1 transcriptional axis regulating thermogenic gene mRNA stability. Our observations of beneficial effects of LHT in human subjects, as well as the close association of the HSF1 SNP with important metabolic traits, demonstrate the potential of LHT for human health (Fig. 1).

In particular, we would like to also discuss about the potential limitations and perspective of the study. Firstly, the difference of LHT compared to other thermogenic cues. Cold is an effective inducer for beige fat activation through elevated sympathetic signals, which leads to the discovery of β-AR agonists in thermogenic activation [5]. Sever burning has also been reported to induce strong browning effects in adipose tissues due to highly activated sympathetic tone [6]. However, we found that LHT did not cause increases in norepinephrine (NE) or cortisol levels in rodents or humans. As expected, LHT increased blood flow in iWAT, but had no impacts on blood flow or gene expression in BAT. This suggests that LHT is likely safe and does not lead to systemic stress that induces sympathetic activation or stress hormone production. However, it is possible that LHT and β-adrenergic signaling may share the same intracellular signal cascade for thermogenic activation, as we have previously shown that cold stimuli could also induce HSF1 activities in beige fat [7]. It would thus be interesting to test whether LHT and cold may have synergistic effects on metabolic improvement.

Secondly, why would hyperthermia induced beige fat activation and thermogenesis, namely why would adipocytes produce more heat in the face of heat stress? Based on HSF1’s best-known function in proteostasis, we believe that mild hyperthermia induces low concentrations of mitochondrial reactive oxygen species (ROS), which act as signaling molecules to initiate a cascade of cellular events. In this scenario, HSF1 activation and PGC1α/UCP1/HSPs induction are activated to remove the increases in ROS in order to maintain protein integrity, ultimately protecting cells and leading to beneficial effects via a process called mitohormesis [8]. Notably, we have revealed that HSF1 regulates Pgc1α as its major downstream effector via multiple layers, including direct transcriptional regulation [7], A2B1-mediated post-transcriptional regulation [9], as well as forming HSF1/PGC1α functional complex for proteostasis [10]. Taking these studies into consideration, and the phenomena that beige adipocytes can sense both heat and cold in a cell autonomous way [5, 9], it is tempting to speculate that beige fat can sense mild heat or cold stress equally and induces HSF1/PGC1α to trigger mitohormesis and its subsequent beneficial effects. Future studies are needed to examine these ideas.

Lastly, the clinical implication of LHT. In our recent study, we only tested the effects of LHT in a small group of patients (*n* = 33) as a proof-of-principle. Longer and larger clinical studies are therefore needed in order to investigate the long-term effects of LHT on metabolic performances in obese, diabetic or aging populations. Additional safe and efficient methods to deliver heat energy or to target HSF1 activation in human beige fat are also warranted. In addition, it is essential to study HSF1 SNPs on energy metabolism in larger cohorts or in cohorts of different races to gain a more comprehensive understanding of HSF1’s role in human metabolic health. In this regard, a humanized knockin mice of various HSF1 SNPs would be desirable in order to provide sufficient genetic evidence and support for future potential gene editing approaches.
